# Trafficking of BK channel subunits controls arterial contractility

**DOI:** 10.18632/oncotarget.22280

**Published:** 2017-11-03

**Authors:** M. Dennis Leo, Jonathan H. Jaggar

**Affiliations:** Jonathan H. Jaggar: Department of Physiology, University of Tennessee Health Science Center, Memphis, TN, USA

**Keywords:** BK channel, nitric oxide, intravascular pressure, vasoconstrictors

Plasma membrane ion channels modulate the physiological functions of virtually all cell types, including vascular smooth muscle cells (myocytes) [1]. Many ion channels are composed of both pore-forming and auxiliary subunits, with the conventional view that these proteins co- assemble intracellularly prior to anterograde surface trafficking of the multi-protein complex. Recent work in our laboratory has shown that surface ion channel subunit composition is flexible and can be modulated to control activity and cellular function in arterial myocytes [2-5].

Membrane potential is a key regulator of arterial contractility [6]. Membrane depolarization activates voltage-dependent Ca^2+^ channels in arterial smooth muscle cells, leading to Ca^2+^ influx, an increase in intracellular Ca^2+^ concentration and vasoconstriction [6]. In contrast, membrane hyperpolarization reduces intracellular Ca^2+^ concentration, leading to vasodilation. Arterial smooth muscle cells express large-conductance Ca^2+^-activated (BK) potassium channels, which are a homotetramer of pore-forming α subunits (BKα) that can couple to auxiliary β1 subunits that modify channel activity [7]. BK channel activation leads to membrane hyperpolarization and vasodilation, whereas BK channel inhibition results in vasoconstriction [6, 7]. BK channels are activated by several different stimuli, including an increase in intracellular Ca^2+^ concentration and physiological vasodilators such as nitric oxide [7]. In contrast, vasoconstrictors, including angiotensin II and endothelin-1, inhibit BK channels [6]. Previous studies have primarily investigated mechanisms that regulate the activity of surface-resident BK channels. In contrast, only recently has evidence emerged that physiological stimuli also control the surface abundance of BK channel subunits to modulate arterial myocyte contractility.

**Figure 1:**
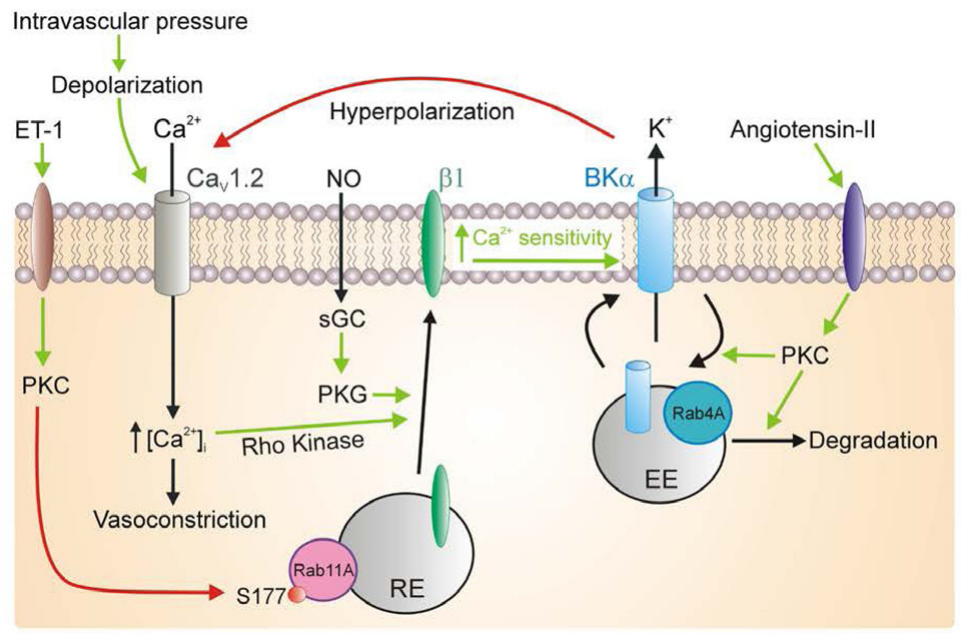
Trafficking of BK channel subunits controls BK channel activity and arterial contractility BKα subunits are surface-trafficked by rab4A-positive early endosomes. Angiotensin II-activated PKC signaling stimulates BKα internalization and degradation. The decrease in BK channel surface abundance leads to membrane depolarization and vasoconstriction. Nitric oxide (NO), through PKG activation, and membrane depolarization, via Rho kinase, stimulate rapid (< 1 min) anterograde trafficking rab11A-positive recycling endosomes that deliver β1 subunits to the plasma membrane. These additional β1 subunits then associate with surface-resident BK channels, increasing their Ca^2+^ sensitivity, leading to an increase in open probability and vasodilation. Endothelin-1 activates PKC which phosphorylates rab11A serine-177 and inhibits surface trafficking of β1, leading to a decrease in BK channel activity and vasoconstriction. Green arrows indicate activation, red arrows indicate inhibition.

The current (I) generated by an ion channel population is the product of the number of channels (N), open probability (P_O_) and single channel current (i), such that: *I=N.P*_*O*_*.i*. Earlier studies focused on identifying mechanisms that control P_O_ in arterial myocytes. In contrast, pathways that regulate the number of ion channel subunits in the plasma membrane (N) remained unclear. Our research in rat and human arterial myocytes was the first to show that BK channel subunit composition is dynamic and modulated by physiological stimuli to control channel activity (Figure 1). In arterial myocytes, BKα subunits are primarily (>95 %) plasma membrane localized, whereas only a small fraction (<10%) of total β1 subunits are present at the cell surface [2]. BKα and β1 subunits are each trafficked by distinct pathways in arterial myocytes. BKα subunits are localized within and surface trafficked by rab4A-positive early endosomes (Figure 1) [3]. In contrast, β1 subunits are stored within rab11A-positive recycling endosomes and are surface-trafficked in response to specific stimuli [2]. We have shown that NO, through the activation of protein kinase G (PKG), and protein kinase A (PKA), stimulate rapid (≤1 min) surface trafficking of β1 subunits which then associate with BKα subunits present at the cell membrane [2]. β1 is constantly recycled and NO stimulates PKG-mediated phosphorylation of rab11A to increase β1 surface abundance (Figure 1). The increase in surface β1 subunits elevates the apparent Ca^2+^-sensitivity of BK channels, leading to activation and vasodilation [2].

Intravascular pressure stimulates arterial depolarization and activates BK channels [6]. It was unclear if membrane potential controlled BK currents by modulating surface trafficking of β1 subunits. Recently, we showed that membrane depolarization stimulates anterograde trafficking and plasma membrane insertion of β1 subunits, which then activate BK channels [4]. Myocyte depolarization, acting via a Ca_V_1.2- and Ca^2+^ influx-dependent mechanism, stimulates Rho kinase (ROCK), leading to Rab11A phosphorylation and β1 surface trafficking (Figure 1) [4]. Both ROCK1 and ROCK2 isoforms are required for this process [4]. ROCK activation increases myosin Ca^2+^ sensitivity and stimulates vascular myocyte contraction [8]. Collectively, these studies illustrate that ROCKs are involved in both positive- and negative-feedback regulation of arterial contractility.

Vasoconstrictors, including endothelin-1 and angiotensin II, stimulate protein kinase C (PKC), which inhibits BK currents in arterial myocytes [6]. It was unclear if vasoconstrictors inhibit BK channels by modulating the surface abundance of BK channel α and β1 subunits. We demonstrated that ET-1 activates PKC, which phosphorylates Rab11A at serine 177, leading to a reduction in Rab11A activity and inhibition of β1 subunit surface trafficking [5]. Through this mechanism ET-1 inhibits BK channels and transient BK currents, leading to vasoconstriction (Figure 1) [5]. Our studies have also shown that angiotensin II stimulates PKC-dependent internalization of BKα subunits, which are subsequently targeted for degradation in arterial myocytes (Figure 1) [3]. This mechanism reduces myocyte BK currents, leading to contraction [3]. Thus, vasoconstrictors can reduce the surface abundance of both BKα and β1 subunits to inhibit BK currents in arterial myocytes and stimulate contraction.

In summary, recent studies have shown that vasoregulatory stimuli can modulate the subunit composition of surface BK channels to control activity and arterial contractility. Our data raise the possibility that the subunit composition of other ion channels in arterial myocytes and different cell types may be similarly modulated to control activity.
